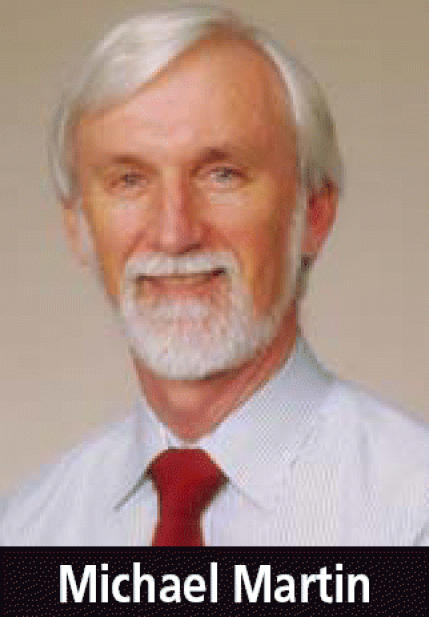# Improving Grant Application Peer Review for the NIEHS

**DOI:** 10.1289/ehp.114-1459941

**Published:** 2006-05

**Authors:** David A. Schwartz, J. Patrick Mastin, Michael Martin

**Affiliations:** Director, NIEHS and NTP, E-mail: david.schwartz@niehs.nih.gov; Chief, Cellular, Organs, and Systems Pathobiology Branch NIEHS, E-mail: mastin@niehs.nih.gov; Director, Division of Physiology and Pathology Center for Scientific Review, E-mail: martinm@csr.nih.gov

The goal of the extramural research program of the NIEHS is to fund the highest-quality science to understand how environmental exposures contribute to the development and progression of human diseases. To identify the most meritorious research proposals, all grant applications submitted to the NIEHS (as well as to other NIH institutes and centers) must undergo individual peer review. These reviews are typically performed by expert committees referred to as “study sections.” Although some of these reviews take place at the individual institutes, approximately 70% of all applications are reviewed at the NIH Center for Scientific Review (CSR). Every year, CSR study sections review tens of thousands of applications, both solicited and investigator-initiated, ranging from small research projects that can be carried out in a short period of time with limited resources to large program projects in areas as diverse as basic molecular biology, nursing research, clinical trials, and bioengineering.

The work of the NIEHS is critical to reducing the burden of disease, improving human health, and extending survival. An improved peer-review process will greatly facilitate our ability to identify and fund the best research to achieve these aims.

Since peer review plays such a vital role in providing information for institutes to use in deciding which proposals to fund, it is crucial that the process ensure the best possible evaluation of the science. NIEHS grantees and applicants have raised concerns over a restructuring at the CSR that brought about changes including the elimination of two study sections dealing mainly with toxicology. In response, members of the NIEHS Division of Extramural Research and Training have embarked on a more formalized and vigorous monitoring and analysis of outcomes of reviews of NIEHS applications by the CSR.

A number of important observations can now be made as a result of this evaluation, which is ongoing and expanding. Our initial evaluation suggests that the CSR restructuring does not appear to have had much of an overall effect on the outcomes of NIEHS applications, although applications in the oncological sciences are not scoring as well since the restructuring, and we are trying to determine if this is true in other topic areas as well. And other problems exist as well. Perhaps the most significant is that NIEHS applications have had less favorable review outcomes than applications assigned to most of the other NIH institutes and centers, in terms of both a larger percentage being “streamlined” (or identified as having less potential for success and therefore afforded a less intensive review process) and a smaller percentage ranking among the highest-scoring 20% of all applications.

There are several factors that may contribute to these outcomes. For instance, the NIEHS has some special characteristics that present challenges to the review of our applications. Our broad scientific mission contributes to applications being dispersed over a wide range of study section topics. We are also relatively small institute. As a result, on average, fewer applications are reviewed for the NIEHS per study section than for most other institutes and centers. Our data indicate that across the NIH, applications from any given institute generally have less favorable outcomes when reviewed in study sections in which they represent less than 5% of the total applications being reviewed. For example, an NIEHS application may be reviewed in a study section dealing with many aspects of a particular disease and one that might therefore be dominated by applications assigned to another institute. In this instance, if the number of NIEHS applications is small, especially if it represents less than 5% of the total, it is likely they would not fare as well. This is true regardless of the institute or center the application is assigned to, although it can have a greater overall impact on smaller institutes. Although it is unclear why this trend exists, as more information emerges from our evaluation we are confident that its impact can be minimized.

Achieving the best review process for all applications is a key part of the mission of the CSR. To this end, senior staff of the CSR and the NIEHS have had a number of discussions with researchers from the extramural community about the evaluation process, and are developing a plan to increase the percentage of NIEHS applications in any given study section. In addition, we will continue to work together to monitor and improve the peer-review process for NIEHS applications across all NIH study sections. The work of the NIEHS is critical to reducing the burden of disease, improving human health, and extending survival. An improved peer-review process will greatly facilitate our ability to identify and fund the best research to achieve these aims.

## Figures and Tables

**Figure:**
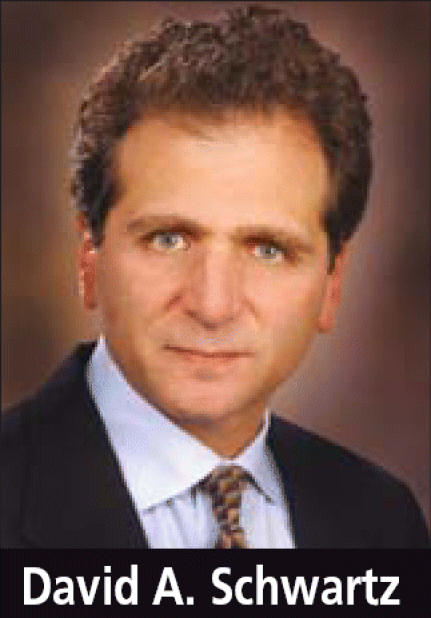


**Figure:**
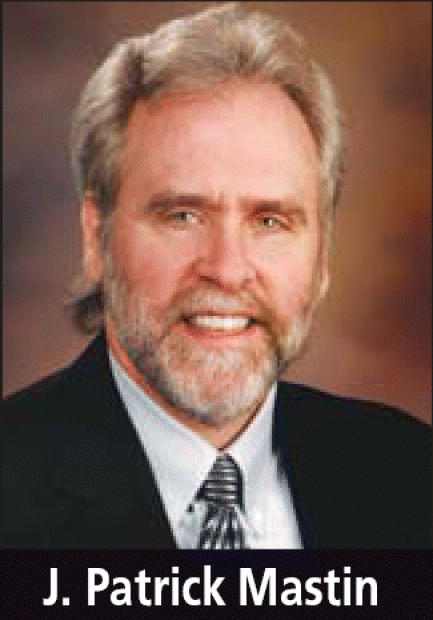


**Figure:**